# The Role of the Cell Surface Mucin MUC1 as a Barrier to Infection and Regulator of Inflammation

**DOI:** 10.3389/fcimb.2019.00117

**Published:** 2019-04-24

**Authors:** Poshmaal Dhar, Julie McAuley

**Affiliations:** ^1^Faculty of Health, School of Medicine, Deakin University, Geelong, VIC, Australia; ^2^Department of Microbiology and Immunology, Peter Doherty Institute, University of Melbourne, Melbourne, VIC, Australia

**Keywords:** MUC1, *Helicobacter pylori*, *Campylobacter jejuni*, influenza A virus, *Streptococcus pneumoniae*, *Pseudomonas aeruginosa*, toll-like receptor, NLRP3-inflammasome

## Abstract

The family of cell surface (cs-) mucins are constitutively expressed at the cell surface by nearly all epithelial cells, beneath the gel-mucin layer. All cs-mucin family members have structural features that enable them to act as a releasable decoy barrier to mucosal pathogens, by providing ligands for pathogen binding and the ability to shed the bound extracellular domain. Due to the towering structure of cs-mucins at the surface, binding of mucosal pathogens can also sterically block binding to underlying cellular receptors. The cytoplasmic tail domain of cs-mucins are capable of initiating signal transduction cascades and due to their conservation across species, may play an important biological role in cellular signaling. MUC1 is one of the most extensively studied of the cs-mucin family. With respect to its physiological function in the mucosal environment, MUC1 has been demonstrated to play a dynamic role in protection of the host from infection by a wide variety of pathogens and to regulate inflammatory responses to infection. This review briefly summarizes the current knowledge and new findings regarding the structural features relating to the function of MUC1, its role as a protective barrier against pathogen invasion and mechanisms by which this cs-mucin regulates inflammation.

## Introduction

To access its receptor on the host cell, invading pathogens must first circumvent the protective mucus layer, which is composed of a network of mucins. Mucins are of two main types, secreted and cell bound. Secretory mucins are synthesized and extruded into the lumen by goblet cells. Cell bound mucins are tethered to the apical cell surface (cs-) of most epithelia and are likely to be the first point of direct contact between host tissues and organisms that penetrate the secreted mucus layer (Hattrup and Gendler, 2008). The first mucin to be characterized structurally was MUC1 and has since been largely studied for its aberrant expression and role in cancer (Taylor-Papadimitriou et al., 1999). However, there is burgeoning evidence that MUC1 plays a dynamic role in the host mucosal barrier to infection. Importantly, MUC1 has been shown to not only provide a physical barrier, limiting infection and colonization, it has also been demonstrated to play a significant role as a modulator of pathogen-induced inflammation (McAuley et al., 2007; Guang et al., 2010; Ng et al., 2016).

**Figure 1:**
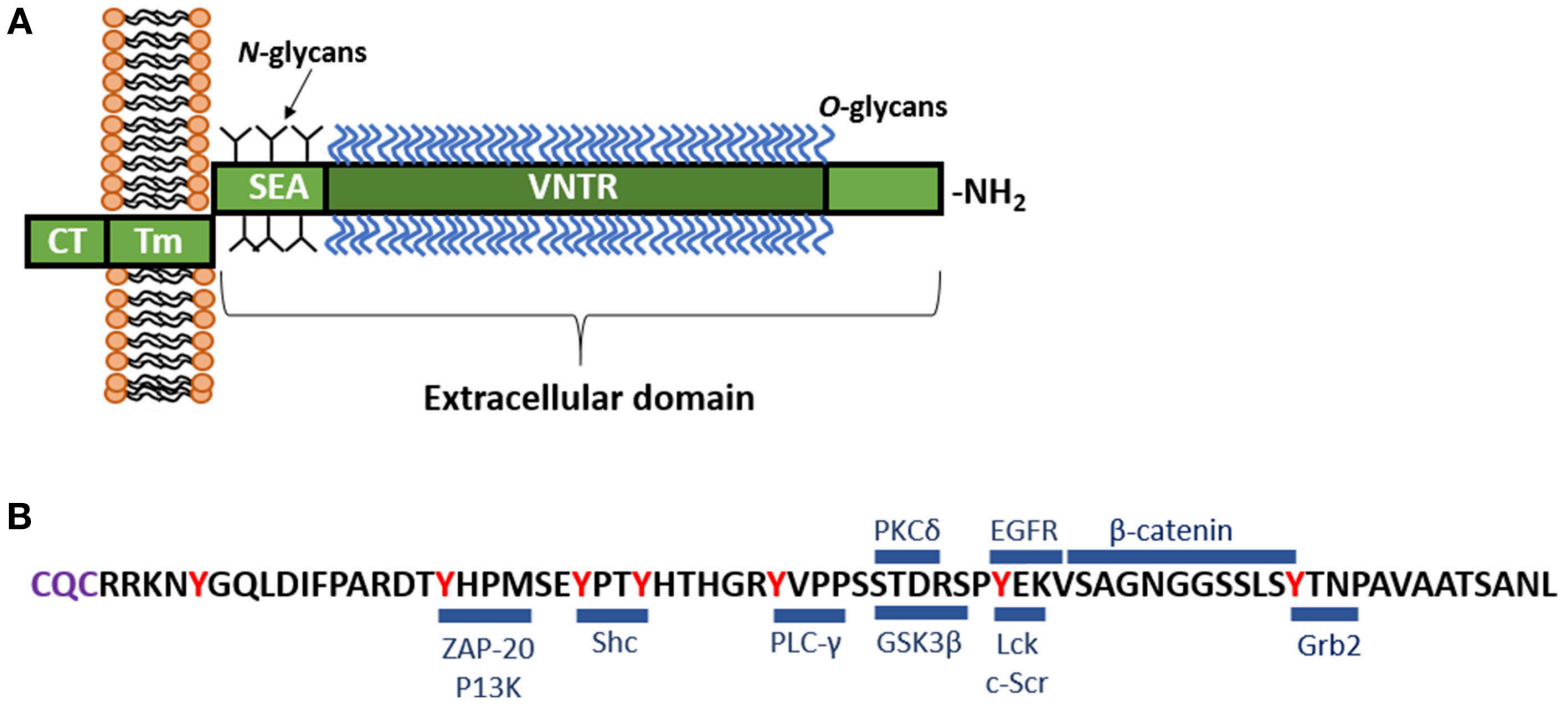
**(A)** Schematic representation of MUC1. MUC1 consists of a large, extracellular, O-glycosylated polypeptide backbone that extends 200–500 nm above the apical surface and consists of the variable number tandem repeat region (VNTR). The VNTR which is composed of between 25 and 125 repeats of a conserved 20 amino acid sequence. This extracellular domain is heavily *0*-glycosylated toward the N-terminal, while closer to the cell membrane, MUC1 displays *N*-glycans. Next to the VNTR lies the sea-urchin sperm protein, enterokinase and agrin (SEA) domain, which is non-covalently linked to the transmembrane (Tm) region and can undergo autoproteolysis in response to a range of stimuli. The Tm region, which is 28 amino acids in length, tethers MUC1 to the cell membrane. Immediately underlying the Tm region is a 72 amino acid long cytoplasmic tail (-CT), which lies inside the cell. The MUC1-CT is known to interact with multiple signaling cascades. **(B)** Amino acid sequence of the MUC1-CT and known binding partners. The MUC1-CT contains 7 tyrosine residues (shown in red), along with serine and threonine residues, which serve as docking sites for kinases and other adaptor proteins. Blue bars mark the known binding sites of specific cellular signaling proteins including: β*-catenin* (Yamamoto et al., 1997); *ZAP-70*, zeta chain-associated protein kinase-70 (Li et al., 2004); P13K, phosphoinositide 3-kinase (Kato et al., 2007); *Shc*, Src homology 2 domain containing protein (Li et al., 2001a); *PLC-*γ, phospholipase C-γ (Wang et al., 2003); *GSK3*β, glycogen synthase kinase 3β (Li et al., 1998); *PKC*δ, protein kinase C-δ (Ren et al., 2002); *Lck*, lymphocyte-specific protein tyrosine kinase (Li et al., 2004); *EGFR*, epidermal growth factor receptor (Li et al., 2001b); Grb2, growth factor receptor-bound protein 2 (Pandey et al., 1995). The C-terminal end of the CT contains the motif CQC (purple) which has been demonstrated to be essential for localization of MUC1 at the cell membrane (Leng et al., 2007).

Similar to all of the cs-mucin family, MUC1 consists of a large, extracellular, O-glycosylated polypeptide backbone that extends 200–500 nm above the apical surface; a transmembrane domain that can be cleaved and enables shedding of the extracellular domain; and a cytoplasmic tail capable of signaling (Figure 1A; Hattrup and Gendler, 2008; Sheng et al., 2012). The MUC1 extracellular domain (-ED) is extensively *O*-glycosylated toward the N-terminus (Figure 1A; Hanisch et al., 1998; Perez-Vilar and Hill, 1999; Silverman et al., 2003) and offers many adhesion sites for microbes (reviewed in Hooper and Gordon, 2001). The extracellular region that is proximal to the cell membrane is *N*-glycosylated which affects folding, secretion and localization of MUC1 (Parry et al., 2006). The glycosylation pattern is influenced by the glycosyl transferases involved in the process of glycosylation, as well as the physiological state and location of the cell (Lloyd et al., 1996; Hanisch and Müller, 2000; Parry et al., 2006).

Characteristic of MUC1 is the presence of a variable number tandem repeat (VNTR) region in the extracellular polypeptide backbone. The MUC1 VNTR consists of 20–125 copies of 20 amino acids GSTAPPAHGVTSAPDTRPAP (Swallow et al., 1987; Ligtenberg et al., 1991; Vos et al., 1991). The VNTR of MUC1 is followed by the sea-urchin sperm protein, enterokinase and agrin (SEA) domain which possess the GSVVV motif, where auto-proteolysis occurs inducing self-cleavage of the molecule. This enables the extracellular domain to be shed from the cell surface, while the transmembrane and cytoplasmic domains remain tethered to the cell membrane (Parry et al., 2001). Proteases, particularly TNF-α converting enzyme, MMP-14, and ADAM17 are capable of inducing release of the MUC1 extracellular domain by acting on the cleavage site in the SEA module (Thathiah et al., 2003; Brayman et al., 2004; Thathiah and Carson, 2004; Macao et al., 2006). Upon cleavage of the SEA module, the extrusion of MUC1 extracellular domain into the lumen provides a mechanism by which the mucin-bound pathogen can be released from the cell and excreted from the body via the mucocilary escalator. As such, this cell surface mucin has been referred to as a releasable decoy barrier to infection (Lindén et al., 2009).

The cytoplasmic tail (-CT) of MUC1 is highly conserved throughout most species (Spicer et al., 1991, 1995; Vos et al., 1991). This region possesses seven tyrosine residues, 4 of which can be phosphorylated by kinases and initiate signal transduction cascades (Figure 1B; Wang et al., 2003). The presence of extracellular EGF-like domains and phosphorylation sites in the cytoplasmic tail suggest MUC1 is able to have a functional role in signaling cascades (Sheng et al., 2012), enabling recycling of the MUC1-ED for re-glycosylation after degradation in the lumen, or as a result of microbial interaction (Altschuler et al., 2000).

## Gastrointestinal Expression of MUC1 Role as a Releasable Decoy Barrier to Infection

*Campylobacter jejuni* is an intestinal pathogen that is a major cause of gastroenteritis in humans (Altekruse et al., 1999). Using an *in vivo* model of infection, MUC1 expression at the epithelial surface was shown to limit gastrointestinal and systemic spread of *C. jejuni* and reduce intestinal inflammation in mice (McAuley et al., 2007). Interestingly, MUC1 deficiency was not found to have an effect on systemic infections caused by another Gram-negative intestinal pathogen, *Salmonella typhimurium*. However, *S. typhimurium* targets Microfold (M) cells, which do not secrete mucins and have a thinner glycocalyx (Sansonetti and Phalipon, 1999), while *C. jejuni* binds intestinal mucins and targets goblet cell thecae *in vivo* (Ruiz-Palacios et al., 2003). In a recent study, MUC1 has been shown to serve as an adhesion receptor for another gastrointestinal pathogen, enteroaggregative *Escherichia coli* (EAEC) and knockdown of MUC1 expression in colonic cancer cell line was also associated with reduced EAEC binding (Boll et al., 2017).

*Helicobacter pylori* are highly motile bacteria that colonize the mucus layer lining the human stomach. *H. pylori* infection is associated with a myriad of pathologies, including gastritis, peptic ulcer disease and severe diseases such as gastric mucosa-associated lymphoid tissue lymphoma and gastric adenocarcinoma (Wotherspoon et al., 1993; Uemura et al., 2001; Malfertheiner et al., 2009). Numerous epidemiological studies have shown an association between *MUC1* VNTR polymorphisms and susceptibility for both *H. pylori*-induced gastritis and gastric cancer. These studies have revealed individuals with short *MUC1* alleles are at a higher risk of *H. pylori*-pathologies which represents a clinical risk factor (Garcia et al., 1997; Silva et al., 2001, 2003; Vinall et al., 2002). The MUC1 extracellular domain contains the antigens Lewis^b^, sialyl Lewis^a^, and sialyl Lewis^x^, which serve as potential binding sites for the *H. pylori* adhesins BabA and SabA (Lindén et al., 2009). Proving MUC1 as a releasable decoy, *H. pylori* binding to MUC1-ED triggered shedding via self-cleavage at the SEA domain, resulting in removal of the bacteria from the cell surface (Lindén et al., 2009). Using murine models of *H. pylori* infection in MUC1-deficient mice (*Muc1*^−/−^), epithelial expression of MUC1 was shown to inhibit the adhesion of *H. pylori* to the gastric mucosae, restricting colonization of the stomach (McAuley et al., 2007; McGuckin et al., 2007; Lindén et al., 2009) and MUC1 expressed by monocytes reduced the severity of *H. pylori*-induced gastritis (Ng et al., 2016).

## MUC1 Influence on the Ability for Pathogens to Infect the Respiratory Tract

*Pseudomonas aeruginosa* causes respiratory infections in humans, particularly in immunocompromised individuals, such as those with cystic fibrosis (Gellatly and Hancock, 2013). Similar to the enteric pathogens *C. jejuni*, EAEC and *H. pylori*, MUC1 serves as an adhesion ligand for the flagellin of *P. aeruginosa* (Lillehoj et al., 2002, 2015). Opposite to the effects of the MUC1 barrier to infection in the intestinal tract, studies reveal a reduced colonization of *P. aeruginosa* in lungs of *Muc1*^−/−^ mice as compared to wild-type (WT) mice (Lu et al., 2006). In the murine *P. aeruiginosa* infection model, reduced colonization of *P. aeruginosa* in *Muc1*^−/−^ mice was due to enhanced clearance via heightened early inflammatory responses to the infection, compared to that observed in WT mice (Lu et al., 2006). Interestingly, the host enzyme, Neuraminidase 1 (NEU1) has been implicated to impede MUC1-mediated protection against *P. aeruginosa* infection (Lillehoj et al., 2015). It was proposed that flagellin of *P. aeruginosa* engagement of NEU1 desialylates MUC1 and other cell surface receptors expressed by airway epithelial cells, unveiling underlying potential *P. aeruginosa* binding mediators, facilitating pathogenicity (Lillehoj et al., 2015). It is unknown whether this NEU1-mediated desialylation of MUC1 occurs in response to other common respiratory pathogens found to interact directly with this cs-mucin.

*S. pneumoniae* are extracellular pneumococcal pathogens that asymptomatically colonize the mucosal surface of the human nasopharynx and upper airways, especially children (Bogaert et al., 2004; Van Der Poll and Opal, 2009). Using the lung epithelial cell line A549 and CRISPR technology to knock-down MUC1 expression, the ability for *S. pneumoniae* to bind to epithelial cells was shown to be MUC1 dependent (Dhar et al., 2017). Furthermore, indicating MUC1 binding to *S. pneumoniae* may play an essential role in triggering phagocytosis, MUC1-deficient macrophages were shown to be inefficient at phagocytosing the pneumococci (Dhar et al., 2017). Dhar et al also demonstrated the inability for mice to express MUC1 was associated with enhanced pneumococcal disease in the murine *S. pneumoniae* infection model.

Influenza A virus (IAV) is a highly contagious respiratory pathogen that is a constant threat to the health of the human population. Recently, it was demonstrated that IAV closely associates with MUC1 and as MUC1 is sialylated, it has the potential to bind to virions, reducing the ability to infect host cells (McAuley et al., 2017). Similar to bacterial infection studies involving the gastrointestinal pathogens *C. jejuni* and *H. pylori*, as well as respiratory *S. pneumoniae, Muc1*^−/−^ mice had heightened inflammatory disease than WT mice caused by the laboratory mouse adapted A/Puerto Rico/8/34 IAV and a 2009 H1N1 pandemic human IAV isolate (McAuley et al., 2017). Linking both barrier and immunomodulatory function of MUC1 during IAV infection, *Muc1*^−/−^ mice reached maximal viral titers earlier than the WT mice, but also exhibited enhanced inflammatory responses to the infection when viral titers were equivalent. Implicating a role for MUC1 barrier and anti-inflammatory function for other respiratory viruses, Respiratory Syncytial Virus (RSV) and human metapneumovirus have both been shown to cause upregulation of MUC1 in a time-course dependent manner in A549 epithelial cells (Li et al., 2010; Baños-Lara Mdel et al., 2015). Li et al additionally showed that during RSV infection of A549 cells caused a negative feedback loop involving TNF production and MUC1 expression, indicating a role for MUC1 in regulating inflammatory disease (Li et al., 2010). Whether MUC1 plays a significant barrier or immunomodulatory role *in vivo* during RSV or pneumovirus infection is currently unknown.

## MUC1 and Regulation of Inflammation During Infection

The innate immune system response mediates the first-line of defense to clear infection and leads to the recruitment of the adaptive arm of immunity. Epithelial and immune cells constitutively express three families of innate pattern recognition receptors (PRRs), which include Toll-like receptors (TLRs), RIG-I-like receptors (RLRs), and NOD-like receptor proteins (NLRPs) that cooperate for early recognition of pathogens and triggering of host responses to infection. It is now clear that MUC1 plays an integral role in regulating inflammatory responses to infection and is a key modulator in controlling pathogen-induced inflammatory disease.

### MUC1 as a Negative Regulator of TLR Signaling

**Figure 2:**
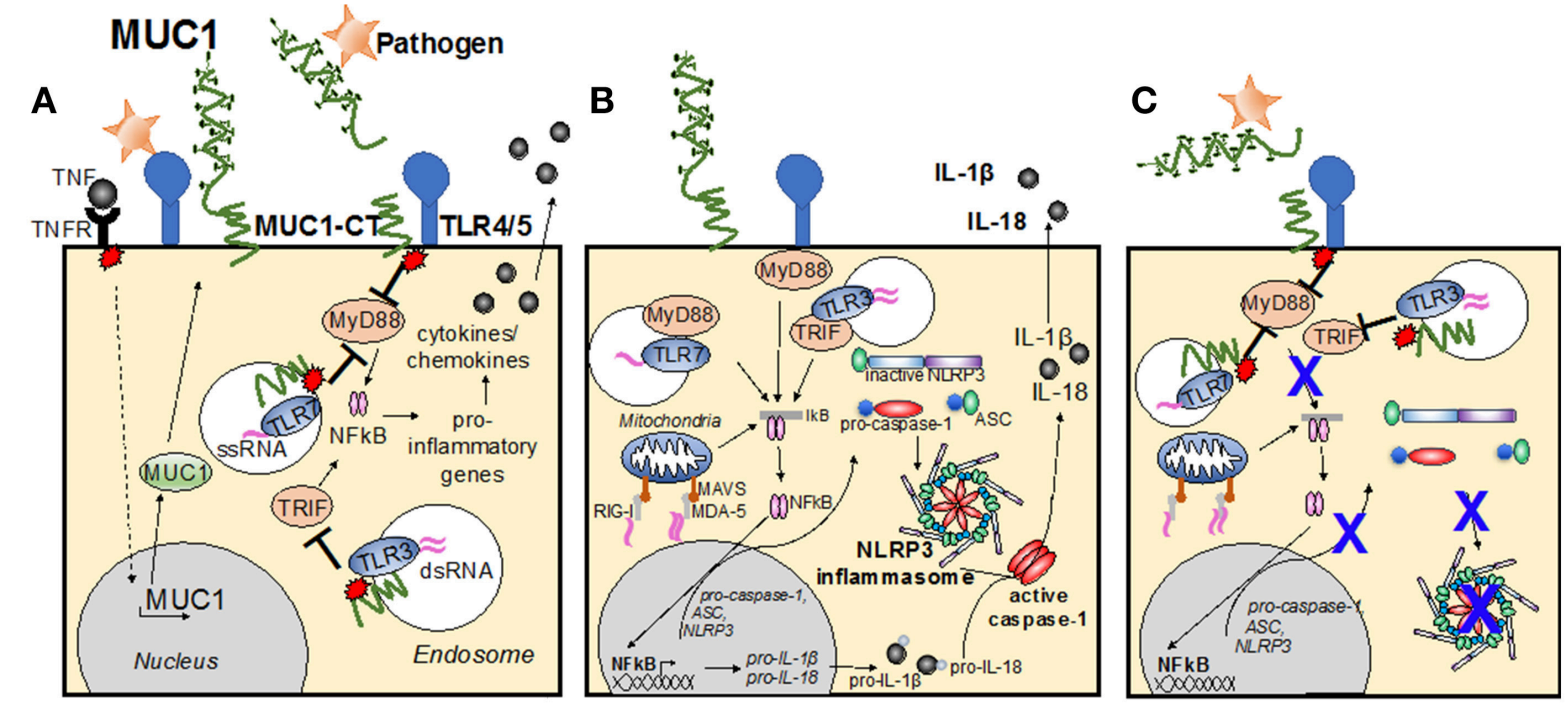
Modes of MUC1 control of cellular inflammatory signaling pathways. **(A)** Infection causes TLR activation, causing production of inflammatory mediators, such as TNF. In turn, MUC1 is upregulated and suppresses TLR signaling, attenuating inflammation. Direct binding of pathogen to MUC1-ED induces phosphorylation of the MUC1 cytoplasmic tail, increasing MUC1-CT association with TLRs at the cell surface, or within the endosome, competitively inhibiting recruitment of MyD88 and/or TRIF to the TLR and blocks downstream signaling. **(B)** Upon detection of PAMPs by PRRs at the cellular membrane and within the endosome, the TLR becomes activated and induces the NF-kB transcription pathway, resulting in upregulation of pro-IL-1β, pro-IL-18 and pro-caspase-1, ASC and NLRP3. A second stimulus then causes NLRP3 to nucleate ASC, inducing the characteristic speck-like structure and causes caspase-1 activation. Active caspase-1 cleaves the pro-IL-1β and pro-IL-18, creating the active cytokines IL-1β and IL-18, which are then secreted from the cell. **(C)** Activation of the MUC1-CT after interaction with pathogen causes recruitment to the TLR, and/or subsequent signaling that limits TLR activation of the signaling cascade and blocks upregulation of inflammasome components. Whether MUC1-CT interacts with NLRP3 directly remains to be determined.

The MUC1-CT has been demonstrated by many studies to play an important role in inhibiting TLR activation. Infection by *Pseudomonas aeruginosa* (Ueno et al., 2008) and respiratory syncytial virus (Li et al., 2010), induces TLR activation in airway epithelial cells and macrophages. This induced production of inflammatory mediators such as IL-8 (KC in the mouse) and TNFα, subsequently recruiting effector cells to the site of infection (Guang et al., 2010; Li et al., 2010; Kato et al., 2016). In turn, MUC1 is upregulated and suppresses TLR signaling, attenuating inflammation (reviewed in Kim and Lillehoj, 2008). It has also been demonstrated that following direct binding of *P. aeruginosa* flagellin to MUC1, the cytosolic epidermal growth factor receptor phosphorylates the tyrosine residues of the MUC1-CT, increasing MUC1-CT association with TLR5, competitively inhibiting recruitment of myeloid differentiation primary response gene 88 (MyD88) to TLR5, and thus blocks downstream signaling (Figure 2A; Kato et al., 2012). MUC1 restriction of the TLR5 pathway further limits downstream signaling cascades, resulting in downregulation of NF-κB-mediated cytokine production (Lu et al., 2006; Kato et al., 2012).

Similar to its suppressive role of the TLR5 signaling pathway, phosphorylation of MUC1-CT has also been implicated as a suppressor of the TLR3-signaling cascade (Figure 2A). Using polyI:C as a model for TLR3 stimulation by double-stranded RNA and murine tracheal, human lung and human embryonic kidney epithelial cells, modified for their expression of MUC1, Kato et al (Kato et al., 2014) demonstrated that activated MUC1-CT co-localizes with TLR3, blocking Toll/IL-1 receptor-domain–containing adapter-inducing IFN-β (TRIF) docking and inhibiting downstream signaling. This reduced the level of apoptosis and production of inflammatory mediators by epithelial cells. Adding credence to the notion that MUC1-CT is a general inhibitor of the TLR pathway, Ueno et al (Ueno et al., 2008) utilized a variety of artificial TLR agonists, including Pam_3_Cys (TLR2), Poly I:C (TLR3), LPS (TLR4), loxoribine (TLR7), and CpG DNA (TLR9), to show that the presence of the MUC1-CT is required for the anti-inflammatory activity of MUC1 during TLR activation. *In vitro* infection studies with non-typeable *Haemophilus influenzae* also revealed that MUC1-CT inhibited the TLR2 signaling pathway (Kyo et al., 2012). Furthermore, links to anti-inflammatory cytokine IL-10 and type I interferon (IFN) correlating with increased MUC1 expression have also been made (Gaemers et al., 2001; Kato et al., 2014). The degree to which MUC1 inhibition of activated TLR pathways diminishes inflammatory responses to infection and the critical timing to which this occurs *in vivo* remains to be elucidated, however must play a significant role in controlling disease severity.

### MUC1 Modulation of the NLRP3 Inflammasome Complex

The inflammasome pathway is now regarded as an essential component of host-defense against infection (Martinon et al., 2002). The inflammasome pathway enables the early detection of pathogens and induces production of inflammatory cytokines, subsequently inducing recruitment of effector cells. The complete mechanism of inflammasome activation requires two signals: an initial priming step upon detection of pathogen (or pathogen-associated molecules), followed by activation of intracellular pattern recognition receptors critical to the formation of the inflammasome complex. For the initial priming step, TLR activation and the associated NF-kB transcription pathway, triggers upregulation of pro-IL-1β, pro-IL-18, and pro-caspase-1, in addition to the inflammasome complex components which include a sensor protein, such as the NLRP, or the receptor protein known as absent in malenoma-2 (AIM2); and the adapter protein known as the apoptosis-associated speck-like protein containing a CARD (ASC) (Figure 2B). Thus, TLRs are the main priming signal for induction of the inflammasome pathway. As such, it is likely that MUC1 is an important inhibitor of the inflammasome pathway, by limiting TLR activation and halting upregulation of inflammasome components (Figure 2C).

Upon priming of signal 2, the NLRPs (or AIM2) cause ASC to nucleate. This causes the clustering of the ASC protein complex, forming a characteristic speck-like structure. The speck then induces oligomerization of pro-caspase 1, inducing it cleavage into the mature form of caspase-1 (Figure 2B; Yang et al., 1998). Active caspase-1 then cleaves pro-IL-1β and pro-IL-18, inducing their activation and secretion, which in turn causes recruitment of effector cells to the site of infection (Martinon et al., 2002).

Production of IL-1β in the stomach potently inhibited gastric acid secretion, reducing the permissible micro-environment enabling *H. pylori* colonization and spread (Wallace et al., 1991; Beales and Calam, 1998). Linking MUC1 as an important suppressor of NLRP3-inflammasome activation was the recent discovery that expression of MUC1 during *H. pylori* infection in mice and *in vitro* suppressed the production of gastric IL-1β (Ng et al., 2016). This was found to in-turn, limit *H. pylori* induced inflammation and resultant pathology of the gastric mucosa (Ng et al., 2016). Furthermore, compared to wildtype macrophages, stimulation of MUC1-deficient macrophages with two other NLRP3-activating bacteria, *H. influenzae* and *S. aureus* have also been associated with elevated levels of IL-1β (Ng and Sutton, 2016). Studies using purified ligands for different inflammasome complexes revealed that this MUC1 restriction is unique for the NLRP3 inflammasome only, as MUC1 deficiency did not have an effect on IL-1β production after stimulation with synthetic activators of other inflammasome receptors (NLRC4, AIM2, and NLRP1b) (Sutterwala et al., 2007; Ng and Sutton, 2016).

Complicating our understanding of MUC1-dependent regulation of the NLRP3 inflammasome, levels of IL-1β in the lungs of *S. pneumoniae* infection of *Muc1*^−/−^ mice may have been attributed to by a lack of appropriate MUC1 signaling, as well as the production of pneumolysin, a toxin produced by *S. pneumoniae* that specifically induces NLRP3-inflammasome activation (McNeela et al., 2010; Witzenrath et al., 2011). Additionally, while influenza infected *Muc1*^−/−^ mice exhibited heightened levels of inflammation (McAuley et al., 2017) and that the NLRP3-inflammasome is considered to be a critical host-response mechanism to the infection (Allen et al., 2009; Thomas et al., 2009), direct links of MUC1 regulation of the complex during influenza infection have yet to be established. Whether the role of MUC1 inhibition of NRLP3-inflammasome dependent inflammation is a general function in response to the majority of mucosal pathogens remains to be elucidated.

## MUC1 as Clinical Markers for Inflammatory Disease

In addition to an association between *MUC1* VNTR polymorphisms and susceptibility for both *H. pylori*-induced gastritis and gastric cancer (Garcia et al., 1997; Silva et al., 2001, 2003; Vinall et al., 2002), altered MUC1 expression levels and glycosylation patterns have been associated with patients with inflammatory bowel diseases such as ulcerative colitis and Crohn's disease (reviewed in Jass and Walsh, 2001). Chronic infections by opportunistic microflora and chronic inflammation of the gastrointestinal tract in those with gastritis, ulcerative colitis and Crohn's disease are hallmarks of the illness severity. While the role of aberrant glycosylation, altered MUC1-ED length and MUC1-regulation of inflammation is far from understood, it is likely that MUC1 does not function appropriately in these individuals and contributes significantly to their disease.

Indicative of a functional role for increased MUC1 expression in respiratory disorders, the sialylated carbohydrate antigen Krebs von den Lungen 6 [KL-6, now identified as human MUC1 (Hirasawa et al., 1997)], is a marker for interstitial lung disease. MUC1 is also elevated in serum taken from patients suffering cystic fibrosis, severe pneumonia, asthma exacerbations, and measles pneumonia (Imai et al., 2002; Ohnishi et al., 2002; Ohshimo et al., 2009). Sputum taken from patients suffering from chronic obstructive pulmonary disease (COPD) also contains significantly more MUC1 compared to samples taken from individuals with prolonged coughing (Ishikawa et al., 2011). With the exception of potential utilization of MUC1 as a diagnostic marker for respiratory disease severity (Ohnishi et al., 2002; Kubota and Haruta, 2006), there has been no study to-date assessing the mechanism(s) of overexpression of MUC1 and the increased vulnerability to inflammatory disease resulting from infection in patients with chronic respiratory conditions.

## Concluding Remarks

Clearly, MUC1 plays a dynamic role in the mucosal barrier to infection and regulation of innate immune responses to control inflammation. Dysregulated and inappropriately glycosylated MUC1 has been correlated with many chronic epithelial diseases and cancers, suggesting when the general function of this glycoprotein is perturbed, normal homeostasis of tissue, cannot be properly maintained. Comprehensively unveiling MUC1-dependent pathways important for maintaining mucosal barrier integrity and modulation of inflammation will aid in our understanding of the development of chronic inflammatory diseases. This will pave the way for development of targeted treatments with the aim to improve mucosal barrier integrity and function, which will ultimately reduce the severity of inflammatory disease, particularly for those suffering chronic conditions.

## Author Contributions

JM and PD contributed equally to the conception and design of the review, as well as development of the figures, manuscript text and revision. Both JM and PD have read and approved the submitted version.

### Conflict of Interest Statement

The authors declare that the research was conducted in the absence of any commercial or financial relationships that could be construed as a potential conflict of interest.
